# Split spiral broadband double channel NMR detector facilitated by LTCC technology

**DOI:** 10.1038/s41598-025-05476-1

**Published:** 2025-06-20

**Authors:** Jianyi Liang, Hossein Davoodi, Khai Chau-Nguyen, Vlad Badilita, Jan G. Korvink

**Affiliations:** 1 Karlsruhe Institute of Technology (KIT), Institute of Microstructure Technology (IMT), Eggenstein-Leopoldshafen, Karlsruhe, 76344 Germany; 2 Littelfuse Inc., Lampertheim, 68623 Germany

**Keywords:** Solution-state NMR, NMR spectroscopy

## Abstract

In the field of nuclear magnetic resonance (NMR), planar spiral detector coils have been exploited for their ease of fabrication with a high tolerance for accommodating flat samples and for the ability to be integrated with microfluidic systems. Additionally, recent studies demonstrated the feasibility of designing such micro planar spirals for broadband operation^1,2^. Here we explore a novel embodiment of the planar spiral with a third contact added at an intermediary position of the spiral track, which thereby acts as a double-channel NMR detector. The spiral geometry was optimized through simulation so that the inner loop and the full loop correspond to two frequency bands of interest. With the combined microfluidic and NMR devices fabricated for the first time in low-temperature co-fired ceramic (LTCC) technology, we demonstrate untuned broadband operation by presenting 1D NMR spectra of 4 isotopes from 125 MHz to 500 MHz, as well as 2D ${^{1}\hbox {H}}-{^{13}\hbox {C}}$ HSQC measurements. The design is easy to use and shows high robustness, demonstrating potential for NMR studies of flow systems with multiple nuclei.

## Introduction

In nuclear magnetic resonance (NMR) spectroscopy, microcoils play a crucial role in achieving high sensitivity, which is a major concern for NMR research. In the first place, the mass sensitivity is inversely related to the coil dimension, such as the diameter in the case of a solenoid coil^3^. Additionally, e.g. in the case of biological tissues, the sample quantity of some chemicals is often limited, due to the high cost involved, or due to availability. In such cases, microcoils provide a high filling factor and thus a higher signal-to-noise ratio (SNR) compared to 5 mm to 10 mm scale coils. Microcoils can also be integrated into NMR spectrometers of different sizes, and flexibly work in cooperation with various setups, such as customized electronics^4^ and microfluidics^5,6^. Coil geometries can be broadly classified into two main categories: volume coils, and planar coils. The former includes solenoid^7,8^, saddle^9^, and Helmholtz coils^10,11^, while the latter includes the spiral coil^12^ and stripline^6,13,14^.

To achieve the expected optimal performance discussed above, microcoils require higher fabrication accuracy and are limited to specific constituent materials. Therefore, multiple microfabrication techniques have been developed for both volume and planar coils. The fabrication process often includes creating an adjacent sample chamber or channel, as an effective RF field is generated close to the microcoil at a sub-cm scale. Walton *et al.* introduced a method to construct a Helmholtz pair by electroplating on glass wafers where two planar spirals were connected by wire bonding, and the sample space was etched at the wafer^10^. Ehrmann *et al.* advanced this by using copper electroplating on wafers combined with an SU-8 microchannel created through photolithography, successfully building both Helmholtz and solenoid coils^11^. Planar circuit boards (PCBs) with FR4 substrates and the complementary metal-oxide semiconductor (CMOS) process have also been investigated for microcoils, providing high fabrication resolution and circuit compatibility^4,15^. Boero *et al.* reported a CMOS-based microcoil with an outer diameter of 150 µm and sample channels fabricated using two-photon polymerization 3D printing with a resolution higher than 1µm^16^. Furthermore, microcoil systems benefit from CMOS technology, which allows for complex integrated circuits for signal amplification and noise control^4,17^, a topic beyond the scope of the present paper.

Glass wafers are widely used as coil substrates because they are non-conductive and do not induce eddy currents. Commercially, glass wafers are available with various thicknesses, roughly ranging from 25 µm to 500 µm, however they are mechanically fragile, and wafer cracks can occur at any step of the fabrication process. This makes processes such as stacking and bonding of glass wafers, for example in order to integrate detector coils with micro-fluidics, rather challenging. Additionally, creating structures with sufficient thickness through etching and deposition is difficult.

As a result, low-temperature co-fired ceramic (LTCC) technology has garnered our attention. LTCCs are sintered from laminated glass-ceramic green tapes, allowing for mechanical structuring, conductor printing on each layer, and through-layer connections. The term “low temperature” refers to sintering processes below ${900}^{\circ }\hbox {C}$. This technology provides a multilayer package with various embedded electrical components, such as resistors and resonators^18,19^. Notably, the substrate material has a low dielectric loss (a loss tangent $<0.002$ at 10 GHz for Ferro A6, for example), making it suitable for reaslizing sensors and microwave devices^20,21^. Open or closed cavities and cantilevers can be engineered with high mechanical strength, high temperature tolerance, and a fabrication resolution down to 10 µm^19,22^. These mechanical structures can serve as fluidic systems and micropumps, among other applications. In terms of NMR applications, LTCC technology was first introduced by Krivic with an LTCC-based micro spiral, though its full potential has yet to be fully explored^23^.

An NMR coil is typically attached to an external tuning and matching circuit that has the role of tuning the resonance frequency of the detector to the Larmor frequency of interest, and ensures impedance matching with a cable, typically 50${\Omega }$. To facilitate the simultaneous detection of different nuclei with high efficiency, multiple-resonance microcoils with corresponding circuits have been developed^24–28^. For example, a single coil equipped with a triple resonance circuit for the simultaneous detection of $^1H$, $^2H$, and $^{13}C$ was reported^24^. Based on a triple-tuned solenoid coil, Collier *et al.* designed a quadruple-resonance probe ($^1H$, $^{13}C$, $^2H$, $^{14}N$) with up to 10 capacitors composed of transmission line segments in the circuit^28^. It is noted that the fine-tuning and matching circuit increases the complexity and cost of building a probe, and also adds to the workload during measurements and daily maintenance.

Instead of using a collection of coils for specific resonance combinations, a broadband coil can cover a wide detection range despite offering moderate sensitivity. The first attempts at broadband NMR probes involved using transmission line coils^29–31^. Kubo and Ichikawa constructed a cascaded low-pass filter with a coil and capacitors, operating from 40 MHz to 300 MHz on a 400 MHz magnet^29^. In another work by Murphree *et al.*, the transmission line probe consisted of a non-resonant solenoid on flexible PCB and was matched to 50${\Omega }$, with an operation range up to 150 MHz where only $10\%$ of the power was reflected from the probe^30^. Compared to resonant detectors, the transmission line probe requires a larger input power for a sufficient pulse length and is up to an order of magnitude less sensitive than common resonant probes^31^.

In the last decade, several broadband micro detectors have been developed and have proven their functionality in high-resolution, high-field NMR^2,32^. These studies have often employed planar spiral geometries, which were previously less favored for resonant detectors. While planar spirals have lower RF field homogeneity compared to solenoid and saddle coils, they offer advantages in coil geometry manipulation, easy fabrication, and flexible integration with microfluidic chips^33,34^. Fratila *et al.* presented a microfluidic glass chip with an untuned single-spiral coil, copper electroplated on a glass wafer, capable of covering a detection range from 61 MHz (${^2\hbox {H}}$) to 400 MHz (${^1\hbox {H}}$) for 1D & 2D NMR measurements at 9.4 T^1^. Using the same microcoil design, recent studies have reported 1D and 2D NMR with $^{19}F$ photo-CIDNP hyperpolarization^32^. Davoodi *et al.* fabricated a spiral coil on a glass wafer and successfully measured seven different isotopes ranging from 126 MHz to 500 MHz^2^. A common feature of these two spiral coils is that they are neither tuned nor matched, but are directly connected to the coaxial cable, differing from the transmission line probes mentioned earlier.

In this contribution, we present an untuned, unmatched, double-port spiral coil design, fabricated using LTCC technology. Introducing a relatively simple but effective modification of the classical spiral geometry, i.e., by splitting the spiral into two segments, we improve sensitivity in two specific frequency ranges for broadband detection. In the first section, we present the simulations of the electrical characteristics of the planar spiral geometry and the $B_1$ field optimization. This is followed by the fabrication of a series of chip devices thinner than 700 µm with an embedded 300 µm high sample channel owing to LTCC technology. We evaluate the fabricated structures and the electrical performance of the LTCC-based detectors. Finally, we report the capability of multinuclear 1D NMR with four different isotopes, as well as ${^{1}\hbox {H}}-{^{13}\hbox {C}}$ 2D NMR with a heteronuclear single-quantum correlation (HSQC) experiment in an 11.7 T magnetic field.

## Results

### Split spiral design and optimisation

**Fig. 1:**
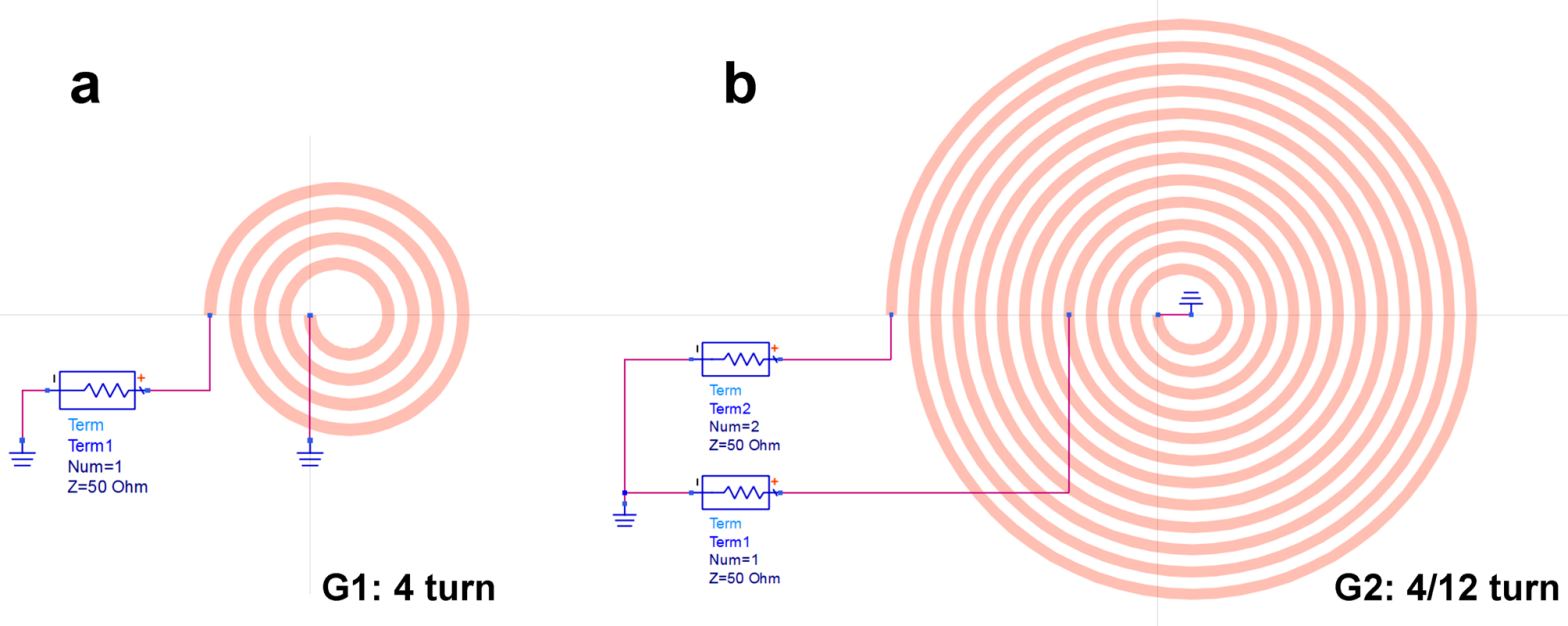
The two spiral models built with the Advanced Design System (**a**) with a single port at 4 turns, and (**b**) with two ports at 4 and 12 turns, respectively referred to as **G1** and **G2** in this work. The spiral model is set to be 100 µm in width and 10 µm in height.

The planar spiral coil is an early member of the first microcoil geometries used as an NMR detector^2,35–37^ due to its ease of fabrication and versatility. The impedance and the resonance frequency of the planar spiral coil can be easily tuned by modifying the number of turns, cross-sectional area of the metal track, loop spacing, and conductor material^2^. It has been shown that such a detector can be successfully used as a broadband detector, albeit with a certain trade-off^1,2^. In a previous review^38^, we have outlined that the frequency range between $\gamma ={0.73}\hbox { MHz}$ per Tesla (${^{197}\hbox {Au}}$) and $\gamma ={16.5}\hbox { MHz}$ per Tesla (${^{7}\hbox {Li}}$) of static magnetic field strength, covers the NMR frequencies of 94% of all active nuclei, whereas the two high-gamma nuclei, ${^{1}\hbox {H}}$ and ${^{19}\hbox {F}}$, very relevant for a wide range of applications, are relatively well separated in frequency with $\gamma ={40.08}\hbox { MHz}$ per Tesla and $\gamma ={42.58}\hbox { MHz}$ per Tesla of static magnetic field strength, respectively. In order to address these two frequency bands in a single broadband detector, we present a subtle modification of the simple one port planar spiral geometry that involves an additional contact along the spiral track, thus transforming the same device into a two-port detector. The concept is presented schematically in Fig. 1 for a 12-turn planar spiral with an additional contact added after 4 inner turns. Throughout the paper, we will denote such a structure as a **4/12** turn two-port detector. The goal of this modification is to use the inner (shorter) spiral to detect high-gamma nuclei, whereas the full spiral is used for the low gamma nuclei.

#### Spiral resonator on LTCC substrate

The electrical behaviour of a spiral resonator built on the LTCC substrate was investigated by conducting a parametric simulation (COMSOL Multiphysics 6.1, Comsol Multiphysics GmbH, Germany) by sweeping the turn number and comparing the frequency and impedance differences.

**Fig. 2:**
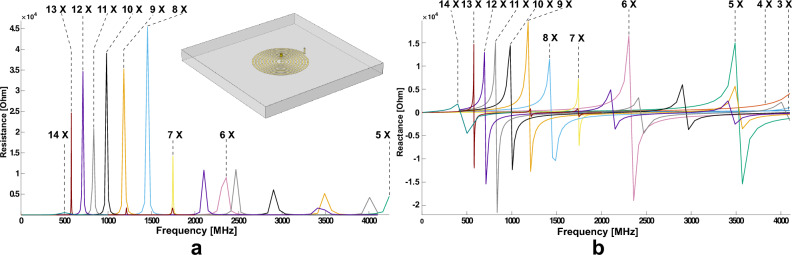
Computational results of the (**a**) real, and (**b**) imaginary parts of the impedance for a single-port G1 spiral with a varying turn number from 3 to 14. Each curve is labeled with the turn number at the first resonance peak. It is noted that the resistances of 3 and 4-turn coils are relatively low at this frequency range and not labeled in (**a**).

The example of simulation model illustrated in Fig. 2 consists of an embedded G1 spiral with 9 turns and a centered via connected to a lumped port with 1 A. A serial simulation of the single channel G1 coil was run with a varying turn number *n* ranging from 3 to 14. The results are shown in Fig. 2 (a) and (b). Fig. 2 is consolidating a straightforward and well-known result: the behaviour of the real and imaginary part of the planar spiral coil impedance as a function of the number of turns, showing that the resonance frequency decreases with increasing the number of turns. The position of the resonance peak exceeds the upper limit of the simulated range of 4 GHz for spirals with $n=3 - 5$ turns, as shown in Fig. 2 (a), while the 6-turn spiral resonates at around 2.4 GHz. The 14-turn G1 spiral has a primary resonance at around 500 MHz. As reported by Anders and Velders^39^, the reactance as the imaginary part of the impedance is almost flat up to around a quarter of the resonance frequency. Thus, a spiral with less than 14 turns in our simulation can ideally detect signals up to 125 MHz while a spiral with less than 12 turns is suitable for 500 MHz. The assumption is based on the result with a spiral alone and is modified with a more complete chip structure design^40^.

#### Optimization of the detector geometry

In this section, we look into the NMR-related characterization of the spiral coils, and optimize the coil geometry towards a high sensitivity and field homogeneity. Davoodi *et al.* built a framework for engineering broadband NMR detectors where two main figures of merits, excitation efficiency and signal-to-noise ratio (SNR), were introduced to evaluate the coil performance at different tuning/matching conditions^2^. We utilized COMSOL Multiphysics to study explicitly the magnetic field distribution of the spiral coils on LTCC substrates.

The effect of the LTCC substrate on the magnetic field distribution is of vital importance in this study, which is enabled by the comprehensive constitutive material assignment in COMSOL. The LTCC substrate is defined with relative permittivity $\epsilon _\text {rel}=7.85$, relative permeability $\xi _\text {rel}=1$, thermal conductivity $\kappa ={4}\hbox {W}\hbox {m}^{-1}\hbox {K}^{-1}$, Young’s modulus $E={120}{GPa}$, and loss tangent $\tan \delta =0.006$, according to the fabrication specification. The spiral and tracks were set to be solid gold.

**Fig. 3:**
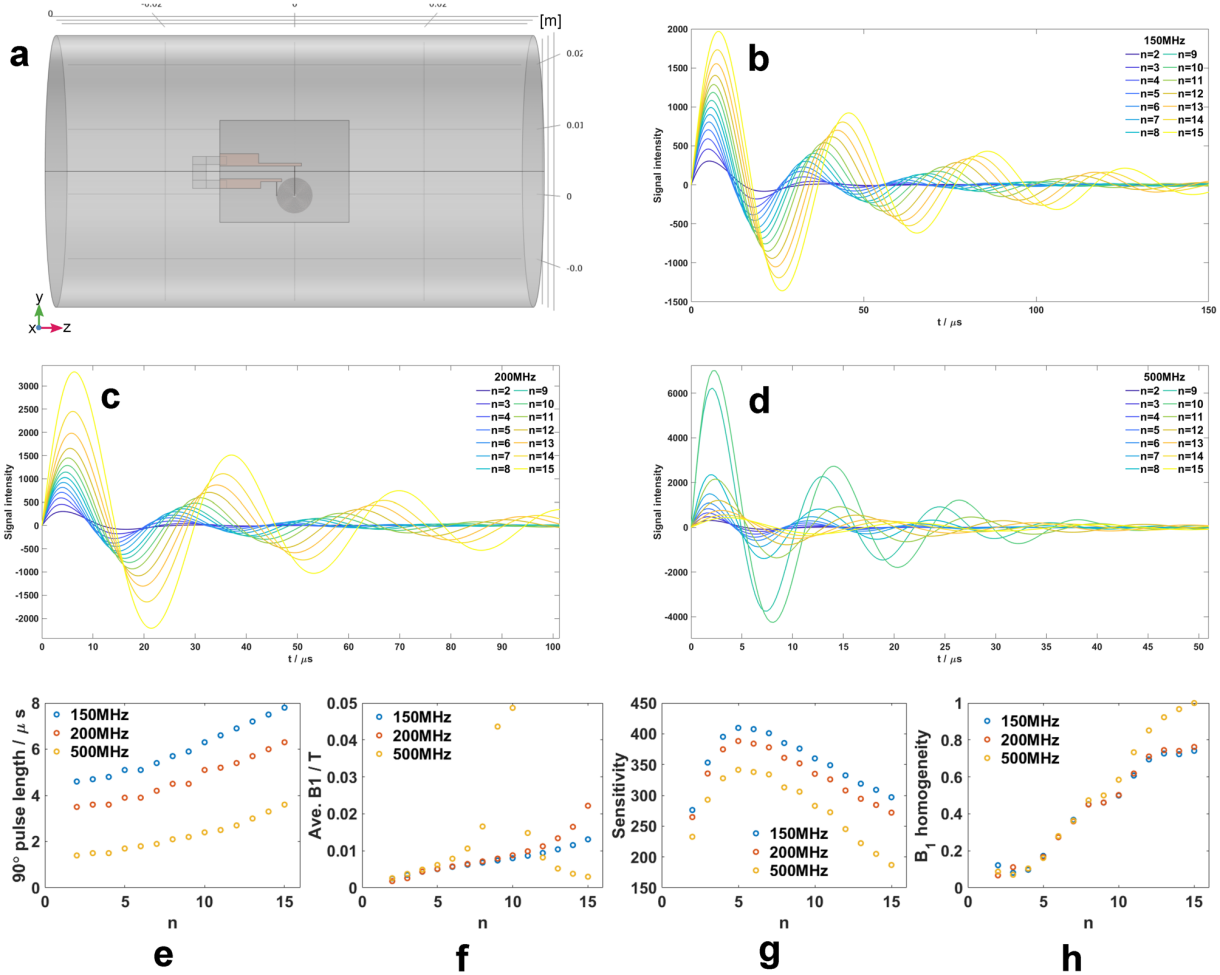
(**a**) Schematic top view of the chip placed at the center of the bore. The conductor structure, including the spiral, is highlighted in orange. (**b**)-(**d**) Simulated nutation spectra for $n \in (2,15)\in \mathbb {Z}$ at three representative frequencies. The time axis is adjusted to reveal flip angle details around $4 \pi$. The following 4 figures are processed from the resulting nutation spectra. (**e**) ${90}^{\circ }$ pulse length. (**f**) Average $\hbox {B}_{1}$ field strength. (**g**) Sensitivity. (**h**) $\hbox {B}_{1}$ field homogeneity.

The embedded coil was connected to conductive pads via through-layer gold vias with a diameter of 150 µm as shown in Fig. 3 (a). A lumped port is added to provide a 1 A source to the coil.

The simulated nutation spectra for $n=2 - 15$ at three frequencies are presented in Fig. 3 (b) to (d) and further processed for a group of figures of merits in (e) to (h). Fig. 3 (e) shows that a higher turn number requires a longer pulse length to realize a ${90}^{\circ }$ flip angle, i.e., more power from the console, and the power consumption is inversely proportional to the frequency. The average $\hbox {B}_{1}$ field in Fig. 3 (f) shows that the field strength benefits from a larger turn number at a low-frequency band, while at 500 MHz it reaches a maximum at $n = 10$ since the coil is close to resonance. The sensitivity in Fig. 3 (g) is evaluated as the ratio of field strength to the square root of the applied power, and the optimal sensitivity is reached by $n = 5$ at all three frequencies. It is noted that the signal region, $r<$1000 µm in diameter, for the sensitivity calculation remains the same for a consistent comparison and thus the filling factor is almost 1 for $n < 6$. As the turn number increases, 150 MHz outperforms the other two frequencies, and the sensitivity difference between 150 MHz and the other two gets larger due to a lower filling factor. Fig. 3 (h) presents the $\hbox {B}_{1}$ field homogeneity normalized with respect to the maximum value for convenient comparison. The RF-homogeneity is calculated by the ratio of the nutation signal amplitudes at ${450}^{\circ }$ and ${90}^{\circ }$. By increasing the turn number, the $\hbox {B}_{1}$ field homogeneity for a fixed sample space is enhanced, and shows little difference between low and high-frequency bands before $n < 10$. From the nutation, the spiral is no longer an effective RF resonator when $n> 10$, but the damping becomes less, making the field homogeneity even higher. Considering these results, we identified 5-turns with the highest sensitivity, and 10-turns as a critical length reaching close to proton resonance, thus selecting the **5/10** G2 spiral for subsequent fabrication and measurement.

#### Resonance behaviour of the optimized split coil

With the split coil geometry established, we did a detailed simulation study of the terminal impedance and the RF field distribution by the split 5/10 coil. The model was further enriched with a sample channel beneath the spiral for a higher simulation accuracy.

**Fig. 4:**
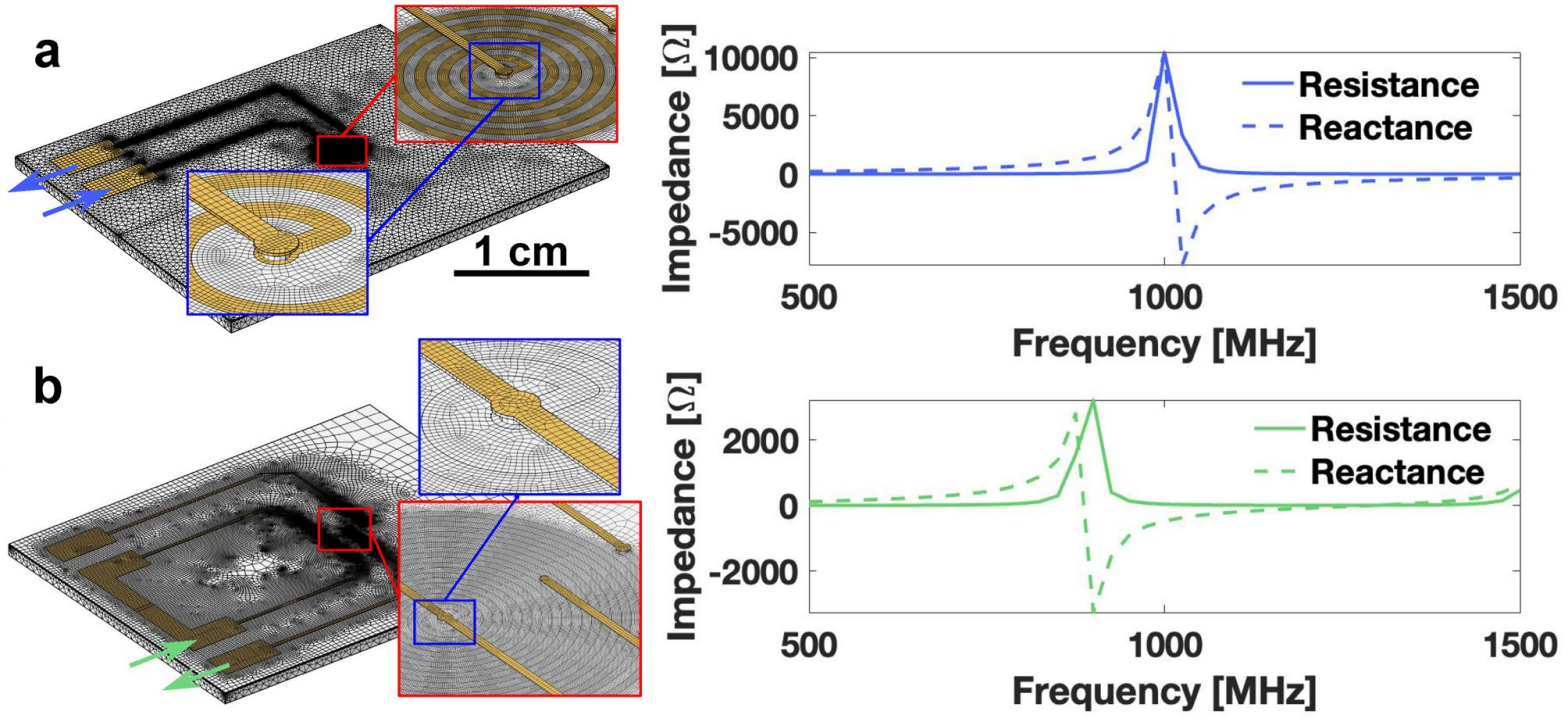
(**a**) Meshed G1-type single 5-turn chip finite element model resulted in ca. 1031k elements and 196k nodes. (**b**) Meshed G2-type 5/10 split spiral chip finite element model resulted in ca. 2353k elements and 460k nodes. In each case the terminal impedance at the port varied from 500 MHz to 1500 MHz.

Fig. 4 compares the self-resonance condition in the inner loop of the split 5/10 coil to that in a simple 5-turn G1 coil. The current density in the coil and the $\hbox {B}_{1}$ field strength (normal to the spiral plane) for 3 working frequencies of the G2 spiral are attached in Fig. S1 in the **Supplementary Information**.

The mesh distribution for two coil models were individually optimized to enhance the simulation precision and decrease the computation time. The results are illustrated in Fig. 4. With the complete chip stack, the self-resonance is decreased due to a higher parasitic capacitance between the substrate and the coil, as the 5-turn G1 spiral possesses a self-resonance at 1000 MHz, and the inner 5-turn spiral of the G2 coil resonates at around 900 MHz, which is around one-third of the resonance frequency of a pure G1 spiral without adding any tracks and pads, as displayed in Fig. 2. The resistances at 500 MHz are respectively 6.0 ${\Omega }$ and 5.1 ${\Omega }$, which results in a similar matching condition with the signal cable.

With the inner port being connected to the 1 A current source and the outer port let as an open circuit, the average current density was lower at the respective frequencies in comparison with the full loop case of a G1 spiral, and the average $\hbox {B}_{1}$ field strength level at the sample region was also lower, as shown in Fig. S1 (a) and (b). Fig. S1 (a) shows the increasing inductive coupling of the outer turns of the 5/10 G2 spiral with the inner, i.e., active turns. This becomes clear in the simulation for 500 MHz. This accounts for the lower self-resonance in Fig. 4 (b). When the outer port was connected to the source, as proven in previous studies^36,41^, the current was more focused in the central segment of the spiral and generated a more homogeneous field with a higher field strength in the sample channel region. Comparing three frequencies, the spiral exhibited the highest excitation efficiency at 200 MHz, which would be beneficial for the application since the full loop should serve a low-frequency range.

### Spiral coil chips based on LTCC technology

**Fig. 5:**
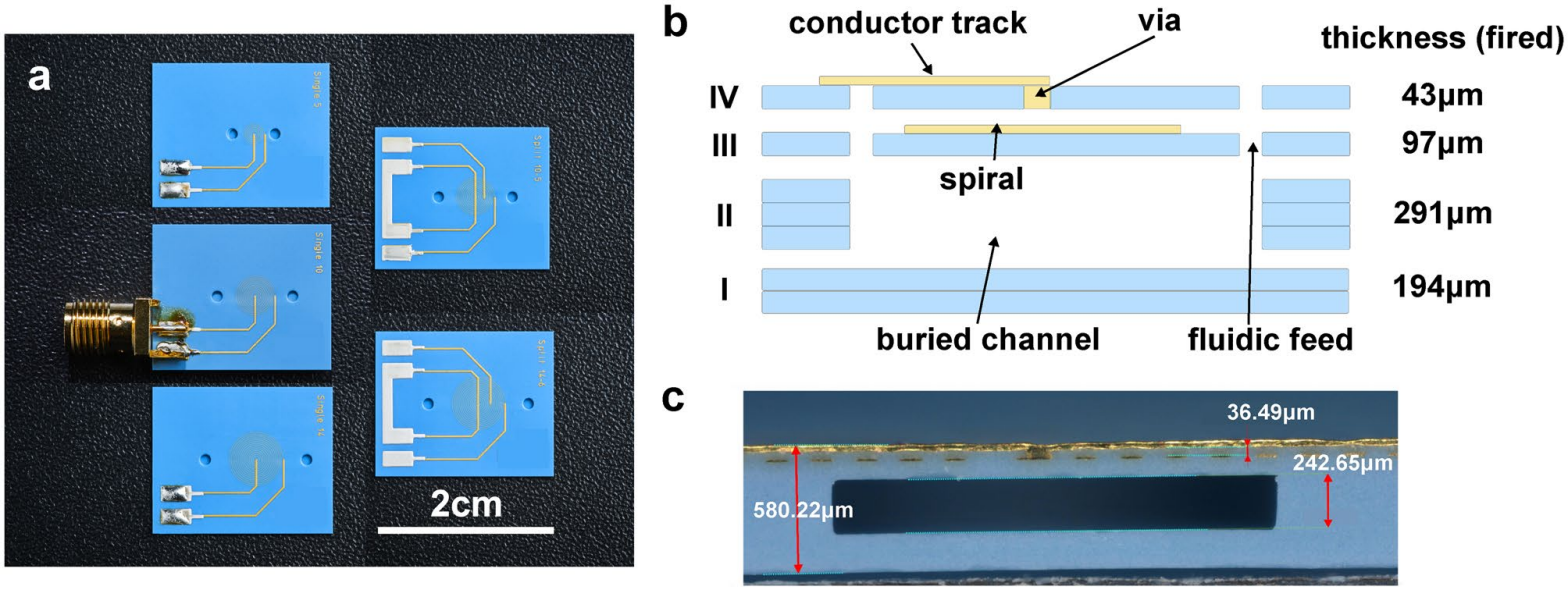
(**a**) Top view of 5 chip devices. The middle device was connected to an SMA connector, for clarification purposes. (**b**) Cross-sectional illustration of the designed low-temperature co-fired ceramic layer stack. (**c**) Correspondingly cut plane of a ceramic chip with annotated principal height dimensions. The dark area within the blue ceramic formed the buried microfluidic channel.

A top view of the five variants of LTCC chips being fabricated is shown in Fig. 5 (a): the 5/10 G2 spiral based on the optimization above, the 5- and 10-turn G1 spiral for comparison, two spirals of respectively 6/14-turn G2 type and 14-turn G1 type being derived from the optimal design to verify the optimization.

The chips are designed in the 7-layer stack structure illustrated in Fig. 5 (b), where two tape thicknesses are used, respectively 43 µm and 97 µm after firing, thus the total chip thickness adds up to around 625 µm. Layer **I** is 194 µm consisting of two 97 µm tapes. The fluidic channel was patterned in the layer **II** using three 97 µm tapes. The 10 µm thick gold spiral was printed on the layer **III** and isolated from the sample channel with LTCC. On the top layer (**IV**), another 10 µm thick conductor layer was printed for conductive pads and tracks, and was connected to the spiral by through-layer vias. The via had a diameter of 150 µm. Inlets and outlets to the buried channel were cut through layer **III, IV** with a diameter of 1.2 mm.

A cross-section image of one fabricated chip is displayed in Fig. 5 (c). The overall thickness was around 0.58 mm and the channel height was around 242.6 µm, which corresponds to a thickness shrinkage rate of around 8%, lower than the 15 % given in the design specification. The warpage on the top LTCC surface was measured to be $< 0.3 \%~mm$. It is noticeable that the two chips with split spirals in Fig. 5 (a) are covered with silver material on the conductor pads, which are fired *AgPt* paste. Throughout the soldering work of the chip with the SMA connector, we found that the gold tracks were easily detached from the LTCC surface and removed when in contact with the heated soldering tips and soldering paste (up to around ${350}^{\circ }\hbox {C}$). *AgPt* paste applied to the original gold proved to be an effective and robust solution.

### Electrical characterization of fabricated chips

**Fig. 6:**
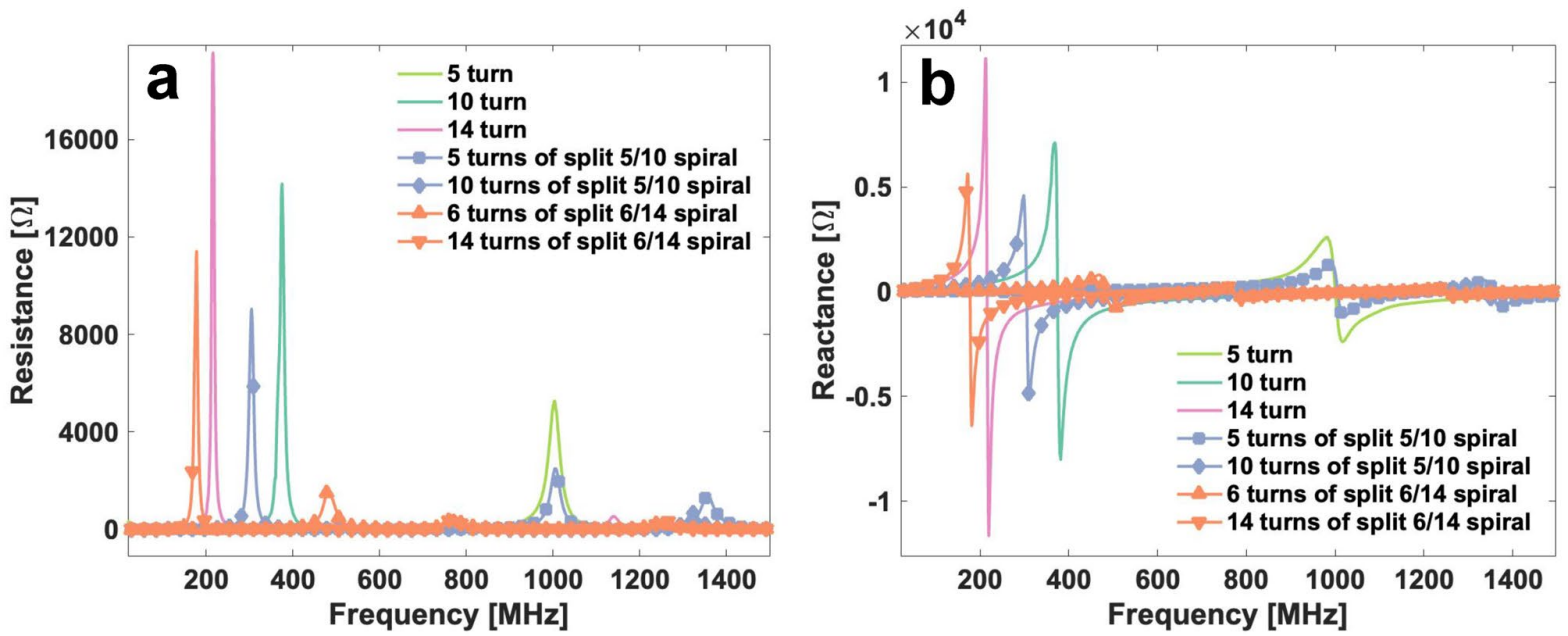
(**a**) Terminal resistance *R* and (**b**) inductance *L* of all chips. The double-port G2 chip was measured such that each port was tested individually, while the other one was left floating. The value of each chip variation was averaged from the raw data collected from several available chip products.

Each fabricated chip was measured with a network analyzer to determine its impedance change, as well as the self-resonance frequency, the results being presented in Fig. 6. For split-spiral chips, two ports are measured independently.

The resonance frequency measured on the 5-turn, 10-, 14-turn G1 spiral is 903 MHz, 369 MHz, and 225 MHz, respectively. According to the untuned broadband chip concept, only a 5-turn spiral is ideal for the detection up to 500 MHz where the other two chips work as a capacitive component. On the split 5/10-turn spiral, the resonance frequency is 307 MHz at the outer port whereas the inner port resonance is higher than 1 GHz. This slightly higher resonance frequency of the inner port of the 5/10-turn G2 spiral than 5-turn G1 spiral can be caused by the double track from the coil center as shown in Fig. 5 since an additional inductor in parallel reduces the total inductance. With respect to the resistance, the inner port of the 5/10-turn chip and the 5-turn chip displayed a close result, 2 ${\Omega }$/2.3 ${\Omega }$ at 125 MHz and 10.7 ${\Omega }$/11 ${\Omega }$ at 500 MHz. The track length was not negligible compared to the spiral total length and the parallel track could also account for the slightly lower resistances on the split spiral coil. On the other hand, the outer port of the split 5/10-turn G2 spiral presented a 60 MHz lower resonance than the G1 spiral. Alhough the double track generated a lower inductance as discussed above, the open track at the 5 th turn contributed a higher parasitic capacitance within the circuit, thus decreasing the resonance frequency. The 14-turn G1 spiral and the 6/14-turn G2 spiral showed a similar phenomenon in the resonance frequency change.

### NMR spectroscopy

#### 1D NMR measurement

**Fig. 7:**
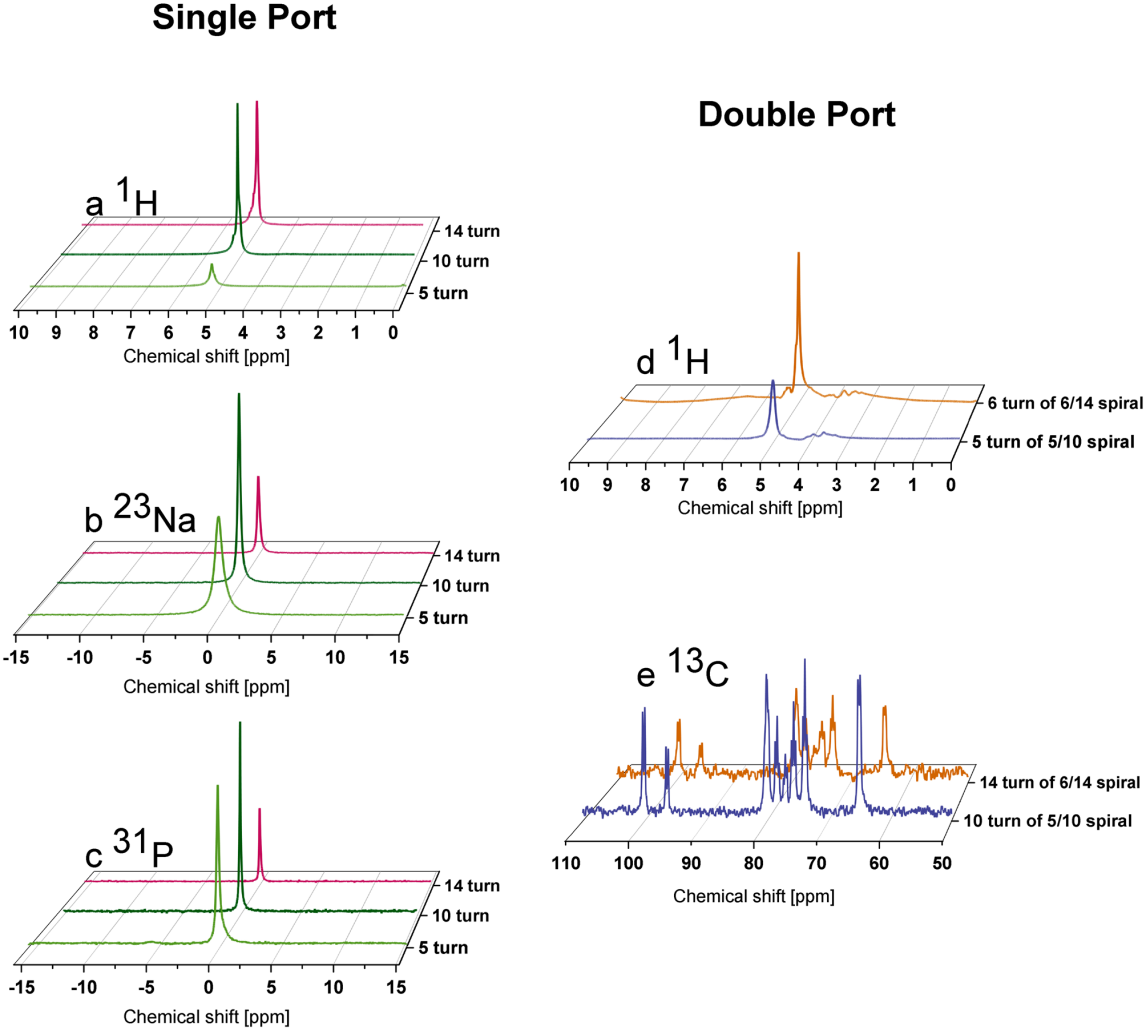
1D NMR spectra of (**a**) $^1H$, (**b**) $^{23}Na$, (**c**) $^{31}P$, from single-port G1 spiral chips, and of (**d**) $^{1}H$, and (**e**) $^{13}C$, from double-port G2 spiral chips.

In this section, we present the 1D NMR measurements with three single-port G1 chips and two double-port G2 chips in broadband configuration. ${^1\hbox {H}}$, ${^{13}\hbox {C}}$, ${^{23}\hbox {Na}}$, ${^{31}\hbox {P}}$ spectra were measured and presented in Fig. 7, covering a Larmor frequency range from 125 MHz to 500 MHz at 11.7 T. Detailed measurement parameters and results are sorted in Table 1 for three G1 chips and Table 2 for two G2 chips, enabling further discussion in a quantitative way.

In three G1 chips, the same solution sample was loaded with 500 mM $Na_2HPO_4$, 250 mM TSP dissolved in 50:50 $H_2O$:$D_2O$. The sample was injected into the channel by a syringe with a soft polymer tube. With a low roughness on the fired LTCC surface, the inlet/outlet was sealed with common laboratory tapes, and the sample was kept in chips for a few days without leakage.

Proton signals in Fig. 7 (a) originated from water and TSP. The water signal peak at 4.8 ppm is picked for the SNR and linewidth calculation. The signal magnitude difference is caused by three factors: the sample volume, scan number and the coil geometry. With the former two contributing factors deprived from the measured SNR, the 10-turn spiral achieved the highest normalized limit of detection (nLOD) as 268 nmol$\sqrt{s}$^42^, i.e., the highest sensitivity among the different coil geometries, and 341 nmol$\sqrt{s}$ by the 5-turn and 989 nmol$\sqrt{s}$ by the 14-turn spiral as shown in Table 1. For each chip, the excitation power level and ${90}^{\circ }$ pulse length documented in the table was optimized in the nutation spectrum and the required transmitted pulse increased with a higher turn number. Compensated by a larger power level, the pulse length ($\tau _{\pi /2}$) got shorter and leads to a wider frequency offset from the carrier ($1/\tau _{\pi /2}$), which is advantageous in terms of measuring all signals at various chemical shifts in a broadband spectrum^4^. The 10-turn spiral also reported the lowest linewidth as 20 Hz, i.e., the best field homogeneity among three single-spiral G1 chips. Despite a high noise level, the 14-turn spiral had a 32 Hz linewidth as half of that by the 5-turn spiral. The sample is exposed to the local static field distortion at the interface between the building material and the sample. For a spiral coil with less turns and accordingly a shorter sample channel, the susceptibility mismatches became more dominant and the linewidths were larger.

${^{23}\hbox {Na}}$ and ${^{31}\hbox {P}}$ each occured as a single peak in Fig. 7 (c) and (d). The maximum sensitivity for ${^{23}\hbox {Na}}$ and ${^{31}\hbox {P}}$ is realized with a 10-turn G1 spiral and a 5-turn G1 spiral respectively. While ${^{1}\hbox {H}}$ and ${^{31}\hbox {P}}$ are both spin-1/2 nuclei and thus not affected by quadrupolar relaxation, ${^{23}\hbox {Na}}$ is a quadrupolar nucleus (spin-3/2) and has very short $T_2^*$, leading to broader lines compared to $^1H$ and $^{31}P$. Same as for the proton case, the 10-turn spiral provided the best $\hbox {B}_{0}$ field homogeneity, 33 Hz at ${^{23}\hbox {Na}}$ and 22 Hz at ${^{31}\hbox {P}}$.

As clearly shown in Fig. S3 in the **Supplementary Information**, limited by its relatively low self-resonance frequency, the sensitivity loss on a 14-turn G1 spiral increased compared to the other two spiral chips as the detection frequency rose from 132.3 MHz to 500 MHz. With the same logic, the 5-turn G1 spiral was supposed to be more sensitive than the 10-turn spiral, which contradicts the data reported above. This is because the 10-turn spiral reaches its resonance at around 500 MHz and the signal gain is enhanced. However, the narrow sensitivity gap between the self-tuned 10-turn and the broadband 5-turn spiral proves the potential of the broadband micro spiral design.

Two double-port G2 spiral chips were also tested for the 1D NMR scenario with a sample of 1M ${^{13}\hbox {C}}$-labeled D-glucose prepared in $D_2O$, i.e., ${^1\hbox {H}}$ from the inner port and ${^{13}\hbox {C}}$ from the outer port, with the results presented in Fig. 7 (d) and (e).

From the ${^1\hbox {H}}$ measurement parameter in Table 2, the inner port of the 5/10 G2 spiral required a ${90}^{\circ }$ pulse of 11 µs as 10 W, which was almost twice that of the 5-turn G1 chip. This additional power consumption was caused by energy dissipation in the outer spiral segment induced by coupling, which was shown in the previous simulation analysis in section **Resonance behaviour of the optimized split coil**. nLOD data was respectively 43 nmol$\sqrt{s}$ and 59 nmol$\sqrt{s}$ by the split 10- and 14-turn spirals, i.e., a 6–8 times ${^1\hbox {H}}$ sensitivity enhancement as the single 5-turn port spiral. The split 14-turn G2 chip reported a signal linewidth of 35 Hz, which was reasonably between the resolutions of the G1-type 5-turn and 10-turn chips. The signal resolution with the 10-turn G1 spiral deteriorated to be 72 Hz, which may have been due to occasional drift of the $\hbox {B}_{0}$ field and an incomplete shimming during the measurement. The high noise level in ${^{13}\hbox {C}}$ measurements was compensated by an exponential line broadening factor of 15. The linewidth was calculated by the leftmost doublet at around 102 ppm, with the split 14-turn chip realizing a linewidth down to 74 Hz.

Based on the characterization above, the split 10-turn G1 chip was proven to have a high broadband sensitivity among the chips fabricated and no notable degradation to the field homogeneity, while the excitation power was still within the range supplied by the electrical system. This experimental result was in good agreement with the simulation prediction and made the 10-turn G2 chip promising for subsequent 2D NMR measurement.

**Table 1:** Broadband NMR measurements at the resonance frequencies of different nuclei, with single-port G1 chips.

Device	5-turn			10-turn			14-turn		
**Isotopes**	$^{\textbf {1}} {\textbf {H}}$	$^{\textbf {23}} {\textbf {Na}}$	$^{\textbf {31}} {\textbf {P}}$	$^{\textbf {1}} {\textbf {H}}$	$^{\textbf {23}} {\textbf {Na}}$	$^{\textbf {31}} {\textbf {P}}$	$^{\textbf {1}} {\textbf {H}}$	$^{\textbf {23}} {\textbf {Na}}$	$^{\textbf {31}} {\textbf {P}}$
Power (watt)	5	10	10	10	20	20	10	25	25
Pulse length (µs)	12	18	15	25	35	20	60	65	50
Number of scans	8	1024	2048	512	128	2048	128	1024	2048
Relaxation delay (s)	10	0.1	5	10	0.1	5	10	0.1	5
Linewidth (Hz)	64	98	50	20	33	22	32	33	34
Measured SNR	703	145	166	13590	527	244	2775	256	150
**nLOD** (nmol$\sqrt{s}$)	**341**	**169**	**120**	**268**	**121**	**214**	**989**	**238**	**378**

**Table 2:** NMR measurements of ${^1\hbox {H}}$ and ${^{13}\hbox {C}}$ with double-port G2 chips. Also see Fig. 8.

Device	**10-turn**		**14-turn**	
Port	Inner # 5	Outer # 10	Inner # 6	Outer # 14
**Isotope**	$^{\textbf {1}} {\textbf {H}}$	$^{\textbf {13}} {\textbf {C}}$	$^{\textbf {1}} {\textbf {H}}$	$^{\textbf {13}} {\textbf {C}}$
Power (watt)	10	30	10	25
Pulse length (µs)	11	25	11	37
Number of scans	32	128	128	128
Relaxation delay (s)	10	1	10	1
Linewidth (Hz)	72	80	35	74
Measured SNR	1563	21	2664	11
**nLOD** (nmol$\sqrt{s}$)	**43**	**1329**	**59**	**1790**

#### 2D NMR measurement

**Fig. 8:**
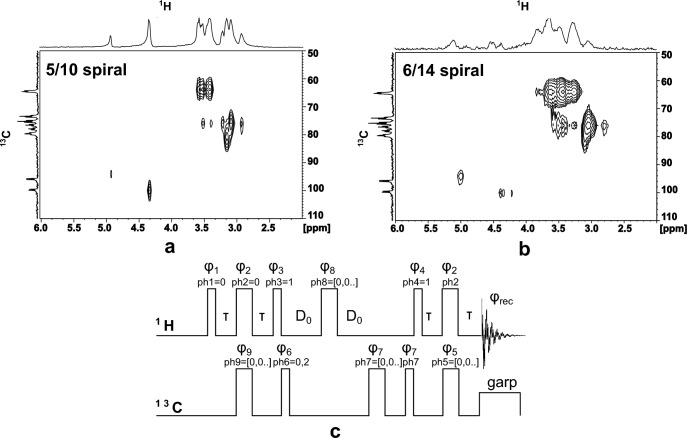
${^{1}\hbox {H}}-{^{13}\hbox {C}}$ HSQC spectra from G2-type (**a**) 5/10 turn and (**b**) 6/14 turn chips, with y-axis representing frequency shift in ${^{13}\hbox {C}}$, and x-axis representing frequency shift in ${^{1}\hbox {H}}$. The required NMR pulse sequences for the two radiofrequency channels are shown in (**c**), with *T* and $D_0$ denoting delays, the $\phi _i$’s refer to the phase angle of each applied RF pulse. A globally optimized alternating phase (garp) excitation pulse decoupled the carbon during proton acquisition. The different pulse parameters applied for each of the two chips are shown in Table 3.

In this section, the implementation of split 10-turn and 14-turn G2 chips are discussed for 2D heteronuclear measurements. Fig. 8 shows the ${^{1}\hbox {H}}-{^{13}\hbox {C}}$ HSQC (Heteronuclear Single-Quantum Correlation) spectra obtained with 1M ${^{13}\hbox {C}}$ labeled D-Glucose dissolved in 10:90 $\hbox {H}_{2O}/\hbox {D}_{2O}$.

The split 10-turn G2 chip provided better sensitivity in the 2D figure than the 14-turn G2 chip comparing Fig. 8 (a) and (b), which is consistent with the 1D NMR results. According to the optimisation process, the split 10-turn G2 chip is an optimum design, while the 14-turn G2 chip, as a deviation variant from the optimal variant, was built alongside this batch as a reference. As a result, the 14-turn G2 chip presented a lower sharpness at the closely spaced carbon signals ranging from 2.8 ppm to 3.7 ppm along the F2 axis (C2/C4 and C3/C5 group when referring to the glucose structure)^43,44^. In both figures, the cross-peaks at around 96 ppm/4.8 ppm and 100 ppm/4.3 ppm on the F1/F2 dimension were clearly resolved, and showed a good separation of the anomeric carbon signals between the $\beta$-D-glucose and $\alpha$-glucose. It demonstrates the capability of the double-port spiral design to be used in molecular structure studies^45^. However, a successful readout of correlated dense ${^{1}\hbox {H}}$ peaks from with closely spaced carbon peaks requires a higher spectral resolution than the current experimental setup.

Two types of pulse sequences were used in the measurement, ${^{1}\hbox {H}}$-decoupled ${^{13}\hbox {C}}$ detection, and ${^{1}\hbox {H}}-{^{13}\hbox {C}}$ HSQC as illustrated in Fig. 8 (c)^46^. The pulse parameters for the experiments are summarized in Table 3. The ${^{1}\hbox {H}}$ (F2 dimension) and ${^{13}\hbox {C}}$ (F1 dimension) sweep widths were 10 ppm and 150 ppm, respectively. In terms of the line broadening factor, we obtained 5 Hz for F1 and 10 Hz for F2.

**Table 3:** Pulse parameters for ${^1\hbox {H}}-{^{13}\hbox {C}}$ HSQC. Also see Fig. 8.

Parameters	5/10 spiral	6/14 spiral
${^1\hbox {H}}$ RF power (watt)	10	10
$${^1\hbox {H}}$$$$\ {90}^{\circ }$$ pulse length (µs)	11	11
Number of data points in FID	4096	4096
${^13\hbox {C}}$ RF power (watt)	25	30
$${^13\hbox {C}}$$$$\ {90}^{\circ }$$ pulse length (µs)	30	28
Number of data points in FID	512	512
Decoupling power (watt)	0.7	0.7
Decoupling length (µs)	20	20
Number of scans	8	8
J-coupling constant *J* (Hz)	145	145
$\tau =(4J)^{-1}$ (ms)	1.72	1.72
Increment delay $D_0$ (µs)	3	3

## Discussion

Facing the challenge of expensive and complicated hardware in NMR systems, the untuned and unmatched planar spiral design is cost-effective and easy to use in conjunction with a flow-arrangement. Without the typical signal processing excitation/reception network on a commercial probe, a simple single-port spiral chip, being connected to the system preamplifier directly, achieved a sensitivity down to 120 nmol$\sqrt{s}$ as shown in this work. Furthermore, a double-port spiral design was introduced featuring two favoured detection ranges, which enhances the sensitivity of the proton measurement by a factor 6, and was capable of 2D NMR measurements for a wider application than a typical spiral detector.

The main challenge of using a micro spiral coil turned out to be the low spectral resolution. The susceptibility mismatch at the interfaces along the static magnetic field direction caused a field distortion, which should be considered when constructing the detector geometry^5,6^. One simple solution to avoid the mismatch would be to move the sample interface away from the coil region, which would inevitably lead to a higher dead sample volume. Another possible reason for the susceptibility mismatch in LTCC chips is the variation of layer thickness and a distorted planarity after firing of LTCC. Symmetric stack design and batch fabrication should be prioritized to avoid severe surface tension difference between top and bottom surfaces. At the same time, the $\hbox {B}_{1}$ homogeneity is of prime importance for the spiral to function as a transmit/receive coil^39^. In our case, the inner loop within a split spiral also recorded the signals from the vicinity of the outer windings. The field uniformity can be improved with a square spiral design and variant turn spacing adjustment^37,41^. One promising direction is to configure the split spiral coil as a TX/RX coil, with the outer loop as the transmitter and the inner loop as the receiver. It could offer potential benefits in tailoring $B_1$ field distribution and optimizing sensitivity for multi-nuclear applications.

Low temperature co-fired ceramics (also denoted as LTCC), as a low-loss substrate material, improved the efficiency and robustness of the coil and sample handling with its high mechanical strength and chemical resistance. It also provides high design flexibility with multi-layer conductor structuring and integrated microfluidic channel. Despite the limitation on minimal conductor width/spacing for printed structures on the LTCC board, as well as layer thickness limitations, an NMR coil based on the LTCC board can be integrated into a miniaturized spectrometer for most micro scale applications. LTCC substrates have dielectric parameters depending on the different recipes for the ceramic compounds, and more LTCC series are emerging^21^. The substrate selection should be guided by the specific application requirements.

In this work, we developed a series of single-port and double-port spiral detectors fabricated using the LTCC technology. The successful execution of both 1D and 2D NMR measurements with a double-port spiral detector demonstrated the effectiveness of this detector design, which extends the application scope of the long-existing planar spiral geometry in NMR detection. With the discussion above, we envision a great potential of the LTCC-based split spiral device in the application to multi-FID detection and in combination with a flow set-up^25,47^.

## Methods

### FEM simulation in COMSOL Multiphysics

COMSOL Multiphysics 6.1 and 6.2 (COMSOL AB, Sweden) was employed with the RF module (Electromagnetic Waves, Frequency Domain) for evaluating the resonance conditions and RF fields of the spiral coils, and optimizing the detector geometry.

The design rules for the devices presented in this manuscript were derived from the materials and process parameters for the LTCC materials released by the manufacturing company (VIA Electronic GmbH, Thuringia, Germany)^48^. We selected the DuPont 951 series for our coil fabrication, since this provided relatively thin-fired LTCC layers (down to ca. 45 µm), thus allowing for more design flexibility in case of multi-level devices. The loss tangent was also relatively low at 0.0063, which is around one-fifth of that for FR4, a widely used PCB substrate. The relative permittivity of 7.85 is among the highest for LTCC materials, providing a good quality factor. The relative permittivity change versus frequency was below 1.6% at a working frequency up to 10 GHz, which was around 5 times less than FR4. The LTCC thermal conductivity of ${4}\hbox {W}\hbox {m}^{-1}\hbox {K}^{-1}$ was also one order of magnitude higher than that of FR4. The conductor pattern in LTCC, either in Au or Ag, had a rectangular cross-section defined by stencil or screen printing. The qualified minimum width of conductor lines and spaces is 100 µm, which is adopted in the designs above. The inner radius of the spirals was set to be 250 µm.

The coil was connected to a lumped port with a 1 A current source. The whole structure was located in a $19 \times 15 \hbox {mm}^{2}$ frame, simulating an NMR chip inside a cylindrical probe chamber, as shown in Fig. 3 (a). The sample in the simulation was assigned as water, as it is one of the most common solvents used in liquid NMR.

The results were processed and illustrated in MATLAB R2018a (MathWorks Inc., USA) for Fig. 2, 3, 4 and S1. The respective processing manuscript is available upon reasonable request.

### Chip layout and fabrication

The layouts of spiral chips shown in Fig. 5 were plotted in LayoutEditor (Juspter GmbH, build: 20190108).

The channel dimensions of each chip variant are presented in Table 4 in detail. In order to have a low field distortion induced by a material boundary close to the detection region^2^, the rectangular sample channel width was set to be half of the outer diameter in the 5- and 10-turn spiral, while the channel length was double the diameter. The split 5/10 spiral took up the dimensions from the 10-turn spiral. The effective sample volume $V_\text {eff}$ was calculated as a box, $2 r \times w \times h$ beneath the spiral layer in the respective cases. The split 6/14-turn spiral chip was adjusted to have the same effective sample volume as the split 5/10 G2 spiral for an SNR comparison.

**Table 4:** Sample channel dimensions of all broadband spiral coil chips.

Device		Channel width *w* [mm]	Channel length *l* [mm]	Eff. sample volume $V_\text {eff}$ [µL]
G1	5-turn	1.20	4.80	0.70
	10-turn	2.00	8.00	2.13
	14-turn	1.40	11.20	2.03
G2	Split 5/10-turn	2.00	8.00	0.97 (5-turn)
		2.00	8.00	2.13 (10-turn)
	Split 6/14-turn	1.40	11.20	0.81 (6-turn)
		1.40	11.20	2.03 (14-turn)

### NMR characterisation settings

The NMR/MRI experiments were conducted on an 11.74 T Avance III Bruker NMR system (Bruker BioSpin, Rheinstetten, Germany). The chips were fixed onto a home-built NMR probe skeleton with a 3D-printed half-cylinder supporter fitting the probe pins as shown in Fig. S1 in the **Supplementary Information**. The chip was held vertically and loaded into the isocenter of the magnet, which is important to avoid local field gradients. The coaxial cable was guided out of the magnet to the pre-amplifier (LNA MODULE 500, Bruker BioSpin, Rheinstetten, Germany) through a copper tube at the center of the probe. No gradient sleeve or matching/tuning circuit were used for NMR measurements, as reported in this paper. The temperature was measured to be ${30}^{\circ }\hbox {C}$ from the system.

For NMR measurement operation and spectra processing, software TopSpin 3.5pl2 was applied. The pulse sequences and the measurement parameters for 1D and 2D NMR are presented with detailed explanation in Sec. **NMR spectroscopy**.

## Supplementary Information

Supplementary Information.

## Data Availability

The data and material that support the findings of this study are available from the corresponding author upon reasonable request.
