# Supplementary material for: Split spiral broadband double channel NMR detector facilitated by LTCC technology
Source: Sci Rep. 2025 Jun 20;15:20162. doi: 10.1038/s41598-025-05476-1 (PMC12181352; doi:10.1038/s41598-025-05476-1)
Supplement: Supplementary file 1 — Supplementary Information. [file 41598_2025_5476_MOESM1_ESM.pdf]

§1 Supplementary Information

§1.1 Simulation of current density and RF field strength in the G2-type spiral

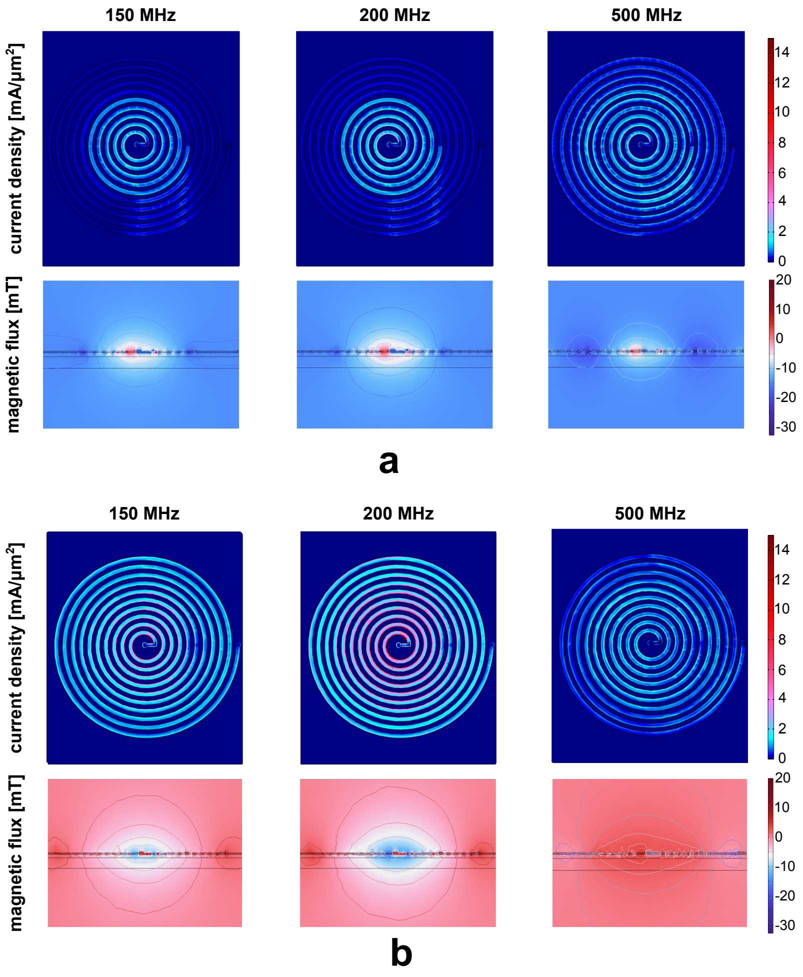

Figure S1: Current density (color map) on each G2-type coil's lateral cross-section and B_1 field strength (color map and contour plot) on the transverse plane with the (a) inner port, or the (b) outer port connected to the terminal. A negative value in the field strength plot refers to a negative B_1 direction.

§1.2 3D printed chip supporter

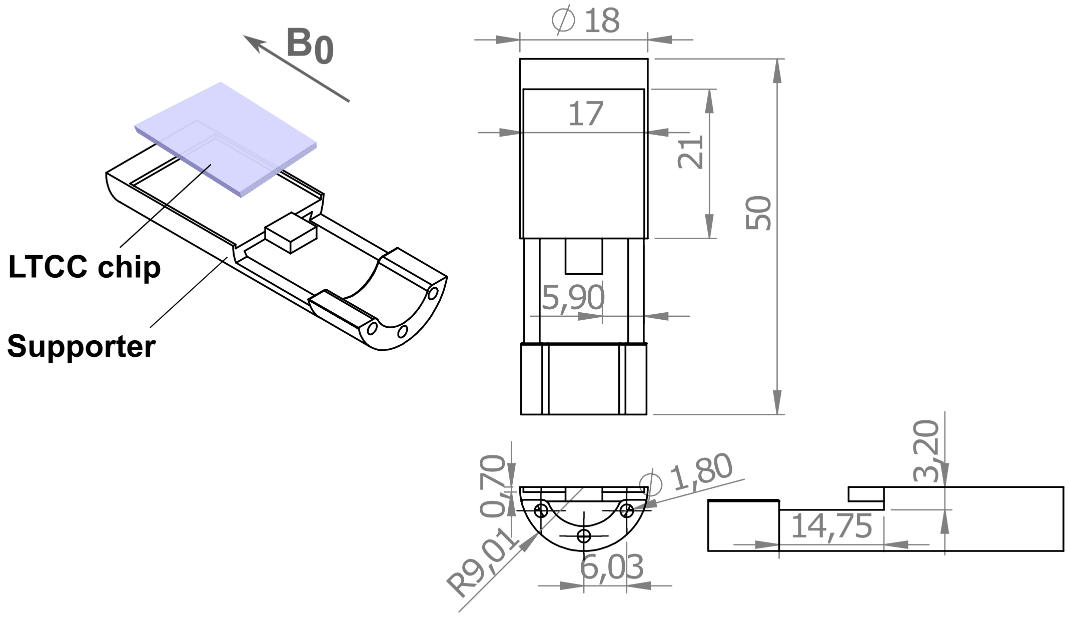

Figure S2: Assembly of the chip model with SLA 3D printed supporter. The dimensional parameters are shown in 3 views in the unit ofmm.

§1.3 Comparison of 1D NMR nLOD

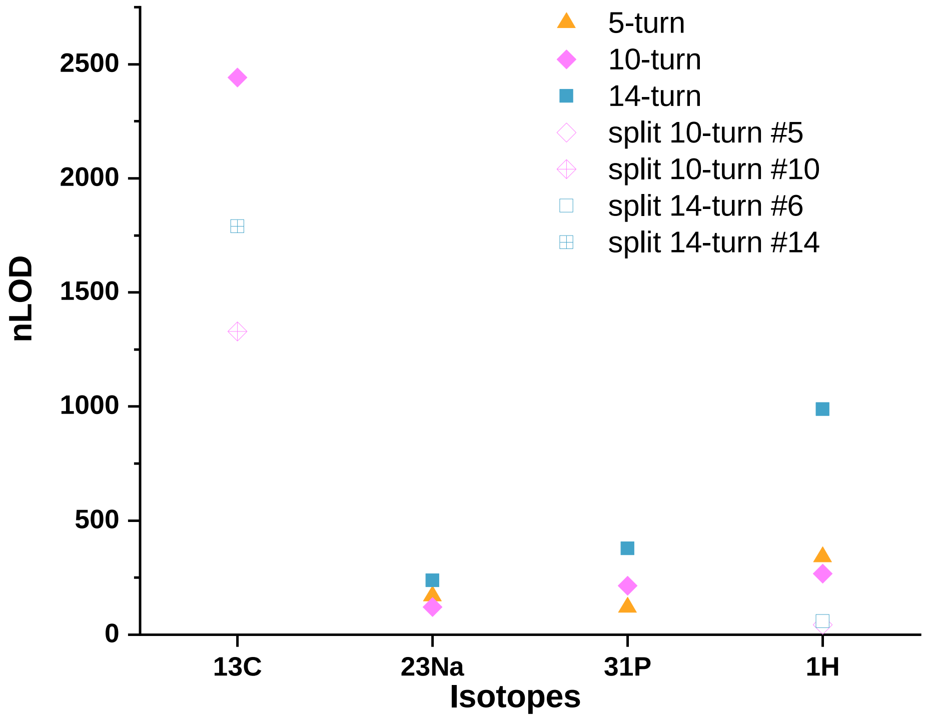

Figure S3: Comparison of the nLOD of 1D NMR measurements using various chips presented in Section **1D NMR measurement**.
